# Neural correlates of working memory in Temporal Lobe Epilepsy — An fMRI study

**DOI:** 10.1016/j.neuroimage.2012.01.126

**Published:** 2012-04-15

**Authors:** J. Stretton, G. Winston, M. Sidhu, M. Centeno, C. Vollmar, S. Bonelli, M. Symms, M. Koepp, J.S. Duncan, P.J. Thompson

**Affiliations:** Epilepsy Society MRI Unit, Department of Clinical and Experimental Epilepsy, UCL Institute of Neurology, Queen Square, London, WC1N 3BG, UK

**Keywords:** Temporal Lobe Epilepsy, Working memory, fMRI, Hippocampus

## Abstract

It has traditionally been held that the hippocampus is not part of the neural substrate of working memory (WM), and that WM is preserved in Temporal Lobe Epilepsy (TLE). Recent imaging and neuropsychological data suggest this view may need revision. The aim of this study was to investigate the neural correlates of WM in TLE using functional MRI (fMRI). We used a visuo-spatial ‘*n*-back’ paradigm to compare WM network activity in 38 unilateral hippocampal sclerosis (HS) patients (19 left) and 15 healthy controls. WM performance was impaired in both left and right HS groups compared to controls. The TLE groups showed reduced right superior parietal lobe activity during single- and multiple-item WM. No significant hippocampal activation was found during the active task in any group, but the hippocampi progressively deactivated as the task demand increased. This effect was bilateral for controls, whereas the TLE patients showed progressive unilateral deactivation only contralateral to the side of the hippocampal sclerosis and seizure focus. Progressive deactivation of the posterior medial temporal lobe was associated with better performance in all groups. Our results suggest that WM is impaired in unilateral HS and the underlying neural correlates of WM are disrupted. Our findings suggest that hippocampal activity is progressively suppressed as the WM load increases, with maintenance of good performance. Implications for understanding the role of the hippocampus in WM are discussed.

## Highlights

► We investigate working memory activity in Temporal Lobe Epilepsy. ► Patients show reduced parietal lobe activity compared to controls. ► Hippocampi progressively deactivate during working memory. ► In patients, only the contralesional hippocampus follows this pattern.

## Introduction

Working memory (WM) refers to the temporary storage and manipulation of information, and is vital for daily life functioning (Baddeley, 2000). Temporal Lobe Epilepsy (TLE) with unilateral hippocampal sclerosis (HS) is associated with significant impairment of the formation and storage of long-term memories (Squire, 1992). In contrast, WM has traditionally been considered to be unaffected by medial temporal lobe (MTL) damage (Cave and Squire, 1992). This contention has been challenged (Ranganath and Blumenfeld, 2005), with evidence of WM dysfunction in TLE (Abrahams et al., 1999; Krauss et al., 1997; Owen et al., 1996; Wagner et al., 2009, for review see Stretton and Thompson, 2012) and for MTL involvement in WM processes (Axmacher et al., 2007, 2008, 2009; Cashdollar et al., 2009). Disruption of WM in TLE may be a result of critical MTL involvement in WM processes (Corcoran and Upton, 1993), or it may be secondary to propagation of epileptic activity from the epileptogenic zone to eloquent cortex responsible for WM function (Hermann et al., 1988). This suggests that the classical functional–anatomical distinctions between long-term memory and WM need to be revised (Cashdollar et al., 2011), and that the cognitive impact of TLE extends beyond episodic memory systems.

The neuroanatomical basis of WM is commonly investigated with variants of the ‘*n*-back’ task (Gevins and Cutillo, 1993). Typically, this requires the monitoring of a series of stimuli, responding whenever a stimulus is presented that is the same as the one presented *n* trials previously (where *n* = 1, 2, 3 etc.). This involves the on-line monitoring, continuous updating and manipulation of information. A meta-analysis of 24 functional MRI (fMRI) data sets for variants of the *n*-back paradigm found consistent activation of bilateral frontal and parietal cortical regions (Owen et al., 2005). However, neuroimaging studies have provided conflicting evidence regarding the precise role and function of the MTL in WM.

There is much evidence to suggest the MTL is independent of WM function (Shrager et al., 2008; Talmi, et al., 2005; Tudesco Ide et al., 2010; Zarahn et al., 2005), proposing that damage to the MTL impairs WM performance only when the task depends more on long-term memory processes (Jeneson and Squire, 2011). In contrast, several imaging studies have found hippocampal activation in WM tasks during encoding (Karlsgodt et al., 2005; Mainy et al., 2007) maintenance (Axmacher et al., 2007) and retrieval (Schon et al., 2009). Conversely, there is also evidence to suggest the MTL deactivates during WM. Early studies using PET in patients with schizophrenia (Meyer-Lindenberg et al., 2001) and fMRI in healthy volunteers (Astur and Constable, 2004; Astur et al., 2005) show a bilateral hippocampal deactivation. More recently, during an *n*-back fMRI task, hippocampal deactivations were enhanced as WM load increased (Cousijn et al., 2012) and this has been shown to be relevant to the maintenance of task performance (Hampson et al., 2006).

Imaging studies in TLE patients examining WM are few, yet have shown disrupted WM networks (Campo et al., 2011; Vlooswijk et al., 2011) as well as distinct hippocampal-dependent and hippocampal-independent WM processes (Axmacher et al., 2007, 2008, 2009; Cashdollar et al., 2009). Axmacher et al. (2007, 2008, 2009) collected intracranial EEG (icEEG) recordings from the MTL of TLE patients with varying pathologies while performing a visuospatial WM task. Single-item maintenance was associated with a sustained slow positive increase in the direct current potential, representing a decrease of activity, which became increasingly more active as WM load increased. Functional MRI in healthy volunteers showed an initial deactivation of the left hippocampus, with increasing activity as WM load increased (Axmacher et al., 2007). The inference was that single-item WM is hippocampus-independent, whereas WM for multiple items is hippocampus-dependent. Similarly, using magnetoencephalography (MEG) there is evidence to suggest theta phase coupling of the MTL and frontal lobes is specifically related to maintenance of configural–relational information in WM (Fuentemilla et al., 2010; Poch et al., 2011). In TLE, maintenance of configural–relational information has been shown to be impaired in patients with bilateral HS relying upon hippocampal-dependent theta networks, while non-configural–relational WM maintenance was hippocampal-independent and preserved in those patients (Cashdollar et al., 2009).

The precise role of the MTL in WM is ill-defined and WM function in TLE remains poorly understood. To date, sample sizes have been small and TLE pathologies have been heterogeneous. To further assess WM in TLE, we employed a visuo-spatial ‘*n*-back’ fMRI paradigm in patients with unilateral hippocampal pathology and healthy volunteers. We aimed to investigate the role of the MTL in WM, and explore whether there is evidence to support hippocampal dependent and independent WM processes. More specifically we hypothesised;

1) WM will be impaired in TLE patients with hippocampal pathology compared to controls.
2) The neuroanatomical basis of WM will be altered in the presence of unilateral hippocampal pathology in relation to laterality.

## Materials and methods

### Subjects

Thirty-eight patients with medically refractory TLE and unilateral hippocampal sclerosis (HS) (19 left, median age 44 years, range 20–56 years, 24 females) undergoing pre-surgical evaluation at the National Hospital for Neurology and Neurosurgery participated in this study. The study was approved by the National Hospital for Neurology and Neurosurgery and the Institute of Neurology Joint Research Ethics Committee, and written informed consent was obtained from all subjects.

All patients had undergone structural MRI at 3 Tesla (3 T). Video-EEG had confirmed seizure onset in the MTL ipsilateral to the HS, and all patients had a normal, contralateral hippocampus based on qualitative and quantitative MRI criteria (Woermann et al., 1998). All patients were taking anti-epileptic medication, were native English speakers and had undergone a neuropsychological evaluation as part of presurgical investigations. Clinical and demographic data are detailed in Table 1.

We also recruited 15 native English speaking IQ matched healthy volunteers (median age 27 years, range 19–58 years, 11 females) without any history of neurological or psychiatric disease.

### MR data acquisition

MRI studies were performed on a 3 T General Electric Excite HDx scanner. Standard imaging gradients with a maximum strength of 40 mT m^− 1^ and slew rate 150 Tm^− 1^ s^− 1^ were used. All data were acquired using an eight-channel array head coil for reception and the body coil for transmission.

For the fMRI task, gradient-echo planar T2*-weighted images were acquired, providing blood oxygenation level-dependent (BOLD) contrast. Each volume comprised 50 oblique axial 2.4 mm slices (with 0.1 mm gap) covering the whole brain, with a 24-cm field of view, SENSE factor 2, 64 × 64 matrix, and an in-plane resolution of 3.75 × 3.75 mm. Echo time (TE) was 25 ms, and repetition time (TR) was 2.5 s.

### ‘Dot-Back’ fMRI paradigm and data analysis

A modified version of the ‘*n*-back’ task (Callicott et al., 1999; Kumari et al., 2003) was used. Subjects were required to monitor the locations of dots (presentation time: 440 ms; inter-stimulus interval: 1500 ms) within a diamond shaped box on the screen at a given delay from the original occurrence (0-, 1-, or 2-back). There were three 30-s active conditions in total (0-, 1-, and 2-back) presented to subjects five times in pseudorandom order, controlling for any order effect. In total, 15 stimuli were presented in each 30-s active block. Each active condition started with a 15-s resting baseline (The word ‘Rest’ appeared on the screen during this period). Subjects were required to move the joystick corresponding to the correct location of the current (0-back) or previously presented (1-back = previous presentation; 2-back = previous presentation but one) stimulus (chance performance = 25%). On-line accuracy data were determined by joystick movement on every trial with output stating either a correct response, wrong response or no response. Percentage of correct 2 Dot-Back trials was used in subsequent analysis as a measure of performance.

### Data analysis

Imaging data were analysed with Statistical Parametric Mapping (SPM8) (www.fil.ion.ucl.ac.uk) using a two-level random-effects analysis. The imaging time series of each subject was realigned using the mean image as reference, spatially normalised into standard anatomical space (scanner-specific template) using the high-resolution whole brain echo planar image and smoothed with a Gaussian kernel of 8 mm full-width at half maximum.

At the first level, for each subject, trial-specific responses were modelled by convolving a delta function that indicated each block onset with the canonical hemodynamic response function (HRF) to create regressors of interest, one regressor for each block (‘0-back’, ‘1-back’ and ‘2-back’). Each subject's movement parameters were included as confounds, and parameter estimates pertaining to the height of the HRF for each regressor of interest were calculated for each voxel. Contrast images for the main effect of multiple-item WM, ‘2-back’ minus ‘0-back’ and single-item WM, ‘1-back’ minus ‘0-back’ were created for each subject. One further contrast image was created to model areas of increasingly negative blood-oxygen level dependent (BOLD) signal change in response to increasing task demand to observe progressive deactivation (Vollmar et al., 2011). Rest was modelled implicitly, and the ‘0-back’ condition was used for baseline comparison. This condition does not require the manipulation of information within working memory yet controls for visual attention and movement related activity. These contrast images were then used for the second-level analysis.

At the second level of the random effects analysis, the subjects were divided into three groups: healthy controls (HC), left HS and right HS. Analysis of variance (ANOVA) was performed with group as a factor to examine the main effects of each contrast and to highlight regions demonstrating more or less activation in one group compared to another. As there was a significant difference in age between patients and controls, we included age as a regressor of no interest in all analyses. We report all activations at a threshold of p < 0.001, uncorrected for multiple comparisons, if not stated otherwise.

### Neuropsychological measures and analysis

IQ was measured using the revised National Adult Reading Test (Nelson and Willison, 1991) (T1). Three WM span tests were administered to each subject outside of the scanner. Span tasks were selected as they require the continuous updating of WM, are sensitive to the effects of increasing WM load, and have been shown to be reliant on the frontal lobes (Owen, 2000).

#### Digit span backwards

The Digit Span subtest from the WAIS-III (Wechsler, 1997) was administered to each participant and the digit span backwards trials were used as the measure of WM. The participants have to repeat digit strings of increasing length in the reverse order. Digit sequences ranged from 2 to 8 with two trials per sequence. Span size was calculated as the highest digit sequence where both trials were successful (max score = 8).

#### Gesture span

The Gesture Span task (Canavan et al., 1989) requires the subject to copy sequences of hand gestures of increasing length up to 5 gestures. The test ends when participants make two consecutive errors at any given gesture set size or when the maximum of 5 gestures had been reached. The task was repeated with a parallel version immediately after the first version was finished. One point was given for each successful trial. The mean span was calculated across trials and was used for the subsequent analysis (max score = 5).

#### Motor sequences

The Motor Sequences task devised by Canavan et al. (1989) requires a sequence of 3 hand gestures to be repeated in the same order. Ten alternating sequences were administered in total. The test stopped after all 10 trials had been completed. One point was given for each successful trial. The total number of successful trials was used for the subsequent analysis (max score = 10).

### Working memory: composite score

In order to explore the relationship between WM competence and neural activation patterns, and to avoid multiple comparisons, a single measure of WM was derived using a principal component analysis (PCA). Out of scanner scores for digits backwards, gesture span and motor sequences as well as performance from the most demanding 2-back fMRI condition were entered into a PCA. One component with an Eigenvalue of greater than 1.0 was found, explaining 58% of the variance. This component was interpreted as an overall measure of global WM capacity.

The derived WM composite score for each subject was then entered as a regressor of interest into an analysis of covariance (ANCOVA) in order to test for correlations between areas of fMRI activation and subject performance. Neuropsychological data were analysed using PASW-v18 (SPSS; Chicago, IL, USA).

## Results

### fMRI findings

#### Single-item WM

**Fig. 1:**
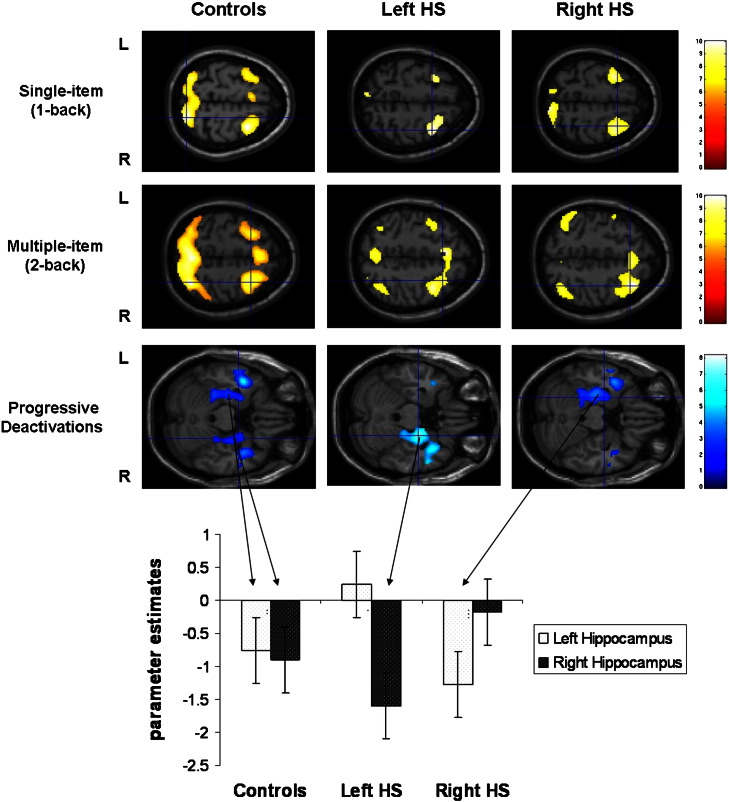
Group results for single-item WM activity (1–0), multiple-item WM (2–0) activity and progressive deactivations. Each group shows significant bilateral fronto-parietal activations (yellow) for single and multiple item WM (p < 0.05 FWE). Progressive deactivation (blue) of the hippocampus was observed bilaterally in controls, but only contralateral to the damaged hippocampus in HS groups (p < 0.01 unc.). The graph depicts the parameter estimates (p < 0.01 unc.) of the negative BOLD signal in the left and right hippocampus of each group. (HS = Hippocampal sclerosis, L = left, R = right).

For the contrast ‘1-back’ minus ‘0-back’ the control group showed significant bilateral superior parietal lobe (SPL) and middle frontal gyrus (MFG) activation. The left and right HS groups showed bilateral MFG activation (Table 2 and Fig. 1). The group comparison revealed both the left and right HS groups had reduced bilateral SPL activity compared to controls. There were no significant differences between patient groups. There was no hippocampal activation as a main effect of task or group.

#### Multiple-item WM

**Fig. 2:**
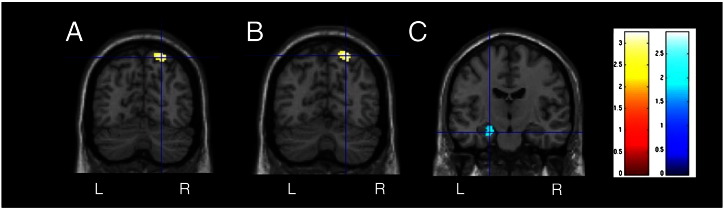
Group comparison between controls and left and right HS patients. Significantly (p < 0.001 unc.) less right superior parietal lobe activation is seen in left (A) and right (B) HS during multiple-item WM (2–0 Dot-Back). C) Reduced progressive deactivation of the left hippocampus (p < 0.05 FWE corrected after 8 mm Small Volume Correction) in the left HS group compared to controls.

Similar to single-item WM, the control group showed bilateral SPL and MFG activity as a main effect for ‘2-back’ minus ‘0-back’. The left HS group showed bilateral MFG, right inferior parietal lobe (IPL) and left SPL activation. The right HS group showed bilateral MFG and bilateral IPL activity (T2 and Fig. 1). Both left and right HS groups showed significantly less activation in the right SPL, compared to controls (unc. p < 0.001) (Fig. 2). There was no hippocampal activation as a main effect of task or group.

#### Progressive deactivations

The controls progressively deactivated the precuneus, superior medial frontal gyrus, superior temporal gyri and bilateral hippocampi in response to increasing task demands (T2 and Fig. 1). The left and right HS groups also showed progressive hippocampal deactivation but only contralateral to the side of the pathology (Fig. 1). The left HS group progressively deactivated the left hippocampus significantly less than the control group (p = 0.029, FWE corrected after Small Volume Correction (SVC) using an 8 mm sphere based on the peak activation, (Fig. 2)). There was a trend toward reduced right MTL deactivation in the right HS group compared to controls though this did not reach significance (p = 0.06, FWE corrected after 8 mm SVC).

### Out of scanner performance

Both left and right HS patients performed less well than controls on all WM performance measures, however only digit span backwards performance reached statistical significance (*F* (2, 52) = 7.5, p < 0.001). An ANOVA of the PCA scores revealed both left (p = 0.01) and right (p = 0.007) HS groups to have a significantly lower global WM capacity compared to controls (Table 3).

### WM composite score regression analyses

In controls, no areas of activation during single-item WM correlated with the WM composite score. During multiple-item WM, better performance was associated with greater deactivation in the left anterior parahippocampal gyrus (− 22, − 24, − 24; z = 3.22, p < 0.001 unc.) Across the task, progressive activation of the right MFG (36, 24, 56; z = 4.05, p < 0.001 unc.) and progressive deactivation of the left medial prefrontal cortex (− 6, 48, 10; *z* = 2.80, p < 0.005 unc.) correlated with better performance.

The left HS group showed greater deactivation of the left inferior temporal gyrus (− 44, 6, − 36; z = 3.37, p < 0.001 unc.) and left anterior hippocampus (− 24, − 14, − 16; z = 2.14, p < 0.02 unc.) associated with better performance during single-item WM. During multiple-item WM, better performance was associated with increased activation of the right MFG (48, 6, 56; *z* = 3.79, p < 0.001 unc.). Across the task, progressive deactivation of the left (− 20, − 36, − 6; *z* = 2.97 p = 0.018 FWE corrected after 8 mm sphere SVC) and right (22, − 38, − 10; *z* = 2.85, p = 0.025 FWE corrected after 8 mm sphere SVC) posterior parahippocampal gyrus and posterior hippocampus was associated with better performance.

The right HS group showed better performance related to increased activation in the right MFG in both single- (40, 36, 30; *z* = 2.74, p < 0.005 unc.) and multiple-item (34, 22, 58; *z* = 2.54, p < 0.005 unc.) WM. Across the task, there was a trend for progressive deactivation of the right posterior parahippocampal gyrus (18, − 38, − 6; *z* = 2.29, p = 0.011 unc.) to be associated with better performance.

## Discussion

We observed disrupted WM in patients with unilateral HS relative to controls. Both left and right HS groups showed reduced right superior parietal lobe activation during the continuous updating of single- and multiple-item WM. With regard to the role of the MTL in WM, our results suggest that the hippocampus is part of the functional network that supports WM. In the presence of unilateral hippocampal sclerosis, this support is disrupted and is specifically related to the side of hippocampal pathology. While in controls, the hippocampi were found to progressively deactivate bilaterally as task demands increased, in HS patients, only the contralateral hippocampus showed progressive deactivation. Regression analysis showed increased activity in the right middle frontal gyrus, and progressive deactivations of posterior MTL structures were associated with better performance.

### Working memory in TLE

Both left and right HS patients performed less well than controls across all measures of WM. This is in keeping with other evidence suggesting WM impairments in TLE (Abrahams et al., 1999; Black et al., 2010; Grippo et al., 1996; Krauss et al., 1997; Owen et al., 1996; Wagner et al., 2009) implying a critical role of the MTL in WM functions. Although not mutually exclusive, two hypotheses for the mechanisms of WM impairment in TLE have been put forward. The first is that propagation of epileptic activity from the epileptogenic zone to eloquent cortex may disrupt cognitive function (Hermann et al., 1988). A recent fMRI study examined WM performance in 36 individuals with cryptogenic focal epilepsy; 10 temporal, 13 frontotemporal and 13 frontal foci based on EEG and seizure semiology. Compared to controls, patients with a temporal pathology were impaired on all measures of WM and showed reduced functional connectivity in a prefrontal WM network comprising the anterior cingulate cortex, middle and inferior frontal gyri. This reduced connectivity was associated with poorer performance on a measure sensitive to the central executive component of WM, with the authors concluding seizure propagation as the cause of disruption (Vlooswijk et al., 2011).

Our data suggest WM related frontal lobe function remains intact in TLE during continuous updating. We observed no significant difference in frontal lobe activity between patients and controls, with all three groups showing increased activation of the right MFG to be associated with better WM. However, we did observed significant reduction of right SPL activity in both patient groups irrespective of side of pathology. Although this region was absent in our regression analyses, it is a robust node associated with continuous updating (Owen et al., 2005), and is critical to the rearrangement and manipulation of information in WM (Koenigs et al., 2009). The reduced SPL activity in HS patients could either be a result of disrupted frontoparietal connectivity, aberrant temporoparietal connectivity, or due to the presence of extratemporal pathology associated with TLE. However, the assessment of parietal lobe function in TLE has not been explored in depth. Two recent studies have reported reduced functional (Liao et al., 2011) and structural (Riley et al., 2010) frontoparietal connectivity in TLE. In addition, a meta-analysis of 18 voxel-based morphometry (VBM) datasets in TLE revealed bilateral parietal atrophy in ~ 50% of the studies (Keller and Roberts, 2008). Similarly, neocortical thinning of bilateral parietal regions was observed in 32 TLE patients with and without HS (Labate et al., 2011). In this context, we interpret our findings as tentative evidence for the propagation of seizure activity from the epileptogenic zone disrupting remote parietal cortex involved in WM.

The second hypothesis for WM impairment in TLE is that the hippocampus is part of the network necessary for WM (Corcoran and Upton, 1993). Our data provides support for this, with reduced performance and disrupted hippocampal involvement in our TLE patients. Similar to previous findings, we did not observe MTL activation during our WM task, but showed progressive deactivation of the hippocampi as task demands increased (Cousijn et al., 2012). In our TLE patients this was seen contralateral to the HS and not in the sclerotic hippocampus. Furthermore, greater WM capacity was associated with greater deactivation of posterior MTL regions across all three groups, however this was more marked in the left hippocampus and parahippocampal gyrus in left HS patients, and to a lesser extent in the right posterior parahippocampal gyrus of the right HS group. Our results suggest that progressive deactivation of the posterior MTL ipsilateral to pathology is crucial to WM task performance in TLE.

In a recent and comprehensive study, Campo et al. (2011) compared dynamic causal models extracted from MEG recordings during verbal WM encoding in 11 left HS patients and 11 healthy volunteers. Using a semantic delayed-match-to-sample task, effective connectivity was modelled using 6 a-priori regions corresponding to the inferior temporal cortex (ITC), MTL, and IFG bilaterally. Twelve models were specified incorporating these nodes using backwards and forward connections unilaterally and bilaterally. The controls performed significantly better than the left HS group and model comparison revealed a bilateral bi-directional model including all nodes yielded the most convincing representation of verbal WM. Comparing this model between groups, the left HS patients showed a reduced ipsilateral backward connection from the left MTL to the left ITC. Contralaterally, patients showed significantly increased forward connections from the right MTL to the right IFG compared to controls, and backward connections from the right IFG and right MTL which were associated with poorer performance (Campo et al., 2011).

While this provides compelling evidence for MTL involvement in WM, unfortunately the parietal lobe was absent from the model selection as this node did not localise during MEG recordings. Thus the authors were unable to comment on the global WM network in TLE and in particular, with relation to the reduced superior parietal lobe activity observed in the current study, the efficacy of temporo-parietal connectivity in unilateral HS patients.

Other studies in TLE have proposed hippocampal-independent and hippocampal-dependent WM systems based on initial deactivation for single-item WM with progressive activation for multiple-item WM (Axmacher et al., 2007, 2008, 2009). Our data is in contrast to this in that we found the hippocampi deactivate to a higher degree for multiple-item WM compared to single-item WM. Axmacher et al. (2007) employed a delayed match-to-sample (DMS) task with 1, 2 and 4 item conditions, in which both encoding and maintenance could be isolated for analysis. In contrast to our task, this places relatively low demands on the continuous updating of WM, placing more emphasis on the temporary storage of items. This enabled the manipulation of higher load conditions (4 items) that could not be applied to a continuous updating task. The level of difficulty would increase to such a degree that it would be detrimental to performance. While both studies highlight a key role of the hippocampus in WM, our results suggest that deactivation of the hippocampus is required for the continuous updating of WM.

Furthermore, recent evidence has suggested that pre-operative fMRI activation in the ipsilateral posterior hippocampus during memory encoding is associated with better episodic memory outcome after anterior temporal lobe resection (Bonelli et al., 2010). Our finding of progressive deactivation of the left posterior hippocampus in association with increased WM capacity would lead us to predict that patients showing this pattern of activation would have preserved WM following anterior temporal lobe resection. We are currently exploring this in those patients with left HS who have undergone anterior temporal lobe resection.

### The role of the MTL in WM

Deactivation of the MTL during WM has previously been observed in healthy volunteers (Astur and Constable, 2004; Astur et al., 2005; Cousijn et al., 2012; Hampson et al., 2006) and has been shown to be reduced in schizophrenia (Meyer-Lindenberg et al., 2001, 2005). Our findings add to this literature, showing the hippocampi and surrounding MTL progressively deactivate as task demand increased with the maintenance of WM performance. Typically task related deactivations have been assumed to be representative of the shift between the neuronal response of task-relevant and task-irrelevant regions implying disengagement of the region, and therefore suspension during cognition (Raichle et al., 2001). However, this disengagement hypothesis does not account for the conflicting reports of hippocampal activation and deactivation during WM.

This discrepancy may lie in the different tasks employed to investigate the neural representations of WM. The two most commonly employed WM paradigms, the ‘delayed match-to-sample’ (DMS) and the ‘*n*-back’, offer competing evidence for MTL activity and deactivation (Cousijn et al., 2012; Olsen et al., 2009). Interestingly, this activation/deactivation has been assumed to reflect hippocampal-dependence (for activation) and -independence (for absence of hippocampal activity or deactivation) (Axmacher et al., 2007, 2008; Cashdollar et al., 2009).

The main difference between the two tasks is that *n*-back paradigms require continuous updating of WM, requiring encoding, maintenance and retrieval of different stimuli at the same time. Using functional imaging, it is difficult to employ an event-related design to disentangle these processes during an *n*-back task. On the other hand, DMS tasks afford the isolation of these independent functions, thereby allowing events to be modelled for each process. Considering the wealth of evidence from the LTM literature implicating the MTL in these processes, it is perhaps not surprising that hippocampal activation is reported in DMS studies. In *n*-back paradigms, as these processes are required to act simultaneously, a hypothesis emerges regarding the deactivation of the hippocampus, in that it may more beneficial to suppress MTL activity that could otherwise interfere with task performance.

In support of this, a recent fMRI study employing the *n*-back paradigm found deactivation of the MTL in healthy volunteers (Cousijn et al., 2012). Investigating the effect of stress on WM, the paradigm employed digit strings using 0- and 2-back conditions. Functional MRI revealed phasic deactivation of the hippocampus, which became more pronounced under stress. Importantly, using arterial spin labelling, they found no detectable change in tonic hippocampal activity under stress, suggesting the deactivations were specifically related to WM processing. The authors argue that stress acts as negative interference akin to task-irrelevant stimuli (Cousijn et al., 2012), and it has been shown that suppression of interference is necessary for the maintenance of optimal performance (Anticevic et al., 2010). Similar to our findings, these results show that dampening of the MTL during continuous updating is functionally relevant in maintaining performance. This implies that WM processes, previously considered hippocampal-independent as a function of regional deactivation, are a necessary and critical aspect of the WM network.

A fundamental question about the neural architecture of WM regards its relationship with other short- and long-term memory systems and whether the hippocampus and MTL are involved in a unitary memory system model (Nee and Jonides, 2008; Oberauer, 2002). Much of the imaging evidence in support of this model has relied upon the observation of hippocampal activation during short-term memory tasks (Nee and Jonides, 2011). However, deactivation of the hippocampus is infrequently reported in LTM and STM encoding and retrieval tasks, thus it has not been discussed in relation to this framework. As the deactivation we observed was functionally relevant in maintaining optimal task performance, we propose the suppression of the hippocampus during WM is crucial, and provides evidence for hippocampal involvement in dynamic memory systems outside that of long-term memory. Our results add to emerging evidence that the functional–anatomical distinction between LTM and WM needs revision (Cashdollar et al., 2011; Ranganath and Blumenfeld, 2005).

### Strengths and limitations

This is the first study to use fMRI to investigate the WM network in a pathologically homogeneous group of individuals with unilateral TLE and HS. Our study does have some limitations. Our fMRI paradigm only assesses spatial WM. While there is support for a large degree of overlap in the neuroanatomical representation of verbal and non-verbal WM (Owen et al., 2005), there is some evidence of material-specific WM impairment in TLE (Wagner et al., 2009). Although we observed no material-specific performance effects of the out of scanner WM span tasks, we plan to acquire data using a verbal version of the *n*-back task to examine the neuroanatomical basis of verbal WM in TLE. Furthermore, our out of scanner assessment of WM was limited. A more extensive WM test battery would help to provide evidence for hippocampal-dependent and -independent processes.

We have not accounted for the influence of anti-epileptic medication and its impact on working memory. There is considerable evidence that topiramate can negatively impact on working memory (Kim et al., 2006; Smith et al., 2006). Few (6) of our patients were taking this drug, though we plan to explore the role of medication further in TLE patients who become seizure free following surgery and discontinue anti-epileptic drug treatment. Finally, while the sclerosed hippocampus has been shown to follow an aberrant pattern of deactivation, it has been suggested that the bold response could be falsely lateralizing related to recent seizure activity (Jayakar et al., 2002). In our cohort this is unlikely, as the interval between the last seizure and time of testing was greater than 24 h in all subjects (T1). Furthermore, any conclusion about the MTL contribution to WM can only be fully assessed with post-operative data following anterior temporal lobe resection.

## Conclusions

This is the first fMRI study to investigate the neural architecture of WM in individuals with TLE and HS. We provide evidence for both local and remote effects of unilateral HS on the functional anatomy of WM. Our data suggest WM is disrupted in those with unilateral HS compared to controls and that progressive deactivation of the hippocampus is required to maintain performance as task demand increases. The progressive contralesional deactivation of the hippocampus relative to increasing task demand is a novel finding in TLE, and highlights the importance of assessing both positive and negative BOLD signal change in cognitive fMRI. Our data also provides further evidence for the critical involvement of the MTL in WM, supporting the notion of task relative hippocampal-dependent WM function and that the MTL has a functional role in memory processes outside of the LTM system.

Furthermore, relevant to our patient sample, functional adequacy of the sclerosed hippocampus at the individual level remains an important clinical target in presurgical investigations to help predict postoperative outcome. The absence of deactivation in the ipsilateral anterior hippocampus in our HS patients may serve as a possible predictor of post-surgical working memory outcome. Whether the altered parietal lobe function is working-memory specific or represents a more global effect of TLE warrants further investigation.

## Figures and Tables

**Table 1:** Group demographics and clinical information.

	Left HS (n = 19)		Right HS (n = 19)		Healthy controls (n = 15)	
Gender (male/female)	10/9		4/15		4/11	
Handedness (left/right)	3/16		3/16		4/25	
	M	IQR	M	IQR	M	IQR
Age (years)	46	11	43	14	27	23
NART IQ	97	12	96	24	105	9
Age at seizure onset (years)	13	21	16	17	n/a	n/a
Duration of epilepsy (years)	22	29	21	30	n/a	n/a
Seizure frequency (per month)	6	12	12	13	n/a	n/a
Interval; last seizure and testing (days)	2.5	9	2	6	n/a	n/a
Average no. of AEDs	2	1	2	1	n/a	n/a

HS; Hippocampal Sclerosis, M; Median, IQR; Interquartile Range, AED; Anti-epileptic medication, NART; National Adult Reading Test.

**Table 2:** fMRI activation peaks for the main effects and interactions of 1–0 dot back, 2–0 dot back, and progressive deactivation contrasts across groups (p < 0.05 FWE corrected across whole brain unless otherwise stated).

Contrast	Group/interaction	Z-score	p-value	Peak coordinates (x, y, z) in MNI space	Anatomical region (BA#)
1-0 Dot back	Control	**5.07**	**0.000**	**20, − 70, 66**	**R. superior parietal lobe (BA7)**
4.63	0.000	32, 2, 56	R. middle frontal gyrus (BA6)
4.82	0.000	− 28, − 66, 62	L. superior parietal lobe (BA7)
4.52	0.000	− 30, 6, 64	L. middle frontal gyrus (BA9)
Left HS	**3.67**	**0.001**	**− 26, 12, 66**	**L. middle frontal gyrus (BA6)**
3.27	0.001	20, 14, 62	R. middle frontal gyrus (BA6)
Right HS	**4.90**	**0.000**	**− 22, 6, 54**	**L. Middle frontal gyrus (BA6)**
4.79	0.000	34, 14, 58	R. superior frontal gyrus (BA6)
4.66	0.000	38, 22, 44	R. middle frontal gyrus (BA6)
Left HS < Controls	**4.02**	**0.001**	**18, − 68, 68**	**R. superior parietal lobe (BA7)**
3.82	0.001	− 30, − 66, 64	L. superior parietal lobe (BA7)
Right HS < Controls	**3.24**	**0.001**	**− 30, − 64, 62**	**L. superior parietal lobe (BA7)**
3.02	0.005⁎	18, − 68, 68	R. superior parietal lobe (BA7)
2-0 Dot back	Controls	**5.50**	**0.000**	**20, − 68, 64**	**R. superior parietal lobe (BA7)**
5.23	0.000	28, 8, 64	R. middle frontal gyrus (BA6)
4.70	0.000	− 30, 6, 64	L. middle frontal gyrus (BA6)
3.93	0.000	34, 24, − 4	R. inferior frontal gyrus (BA47)
3.66	0.000	− 32, 30, − 4	L. inferior frontal gyrus (BA47)
Left HS	**5.38**	**0.000**	**− 6, − 74, 56**	**L. superior parietal lobe (BA7)**
5.36	0.000	20, 10, 66	R. superior frontal gyrus (BA6)
5.35	0.000	− 22 12 68	L. superior frontal gyrus (BA6)
5.13	0.000	− 36, 28, 32	L. middle frontal gyrus (BA6)
4.98	0.000	40, 30, 32	R. middle frontal gyrus (BA6)
Right HS	**5.52**	**0.000**	**− 50, − 56, 54**	**L. inferior parietal lobe (BA40)**
5.29	0.000	40, 26, 40	R. middle frontal gyrus (BA8)
4.94	0.000	− 44, 26, 32	L. Middle frontal gyrus (BA6)
Left HS < Controls	**3.76**	**0.000**	**20, − 68, 66**	**R. superior parietal lobe (BA7)**
Right HS < Controls	**3.34**	**0.000**	**20, − 66, 66**	**R. superior parietal lobe (BA7)**
Progressive deactivations	Controls	**5.29**	**0.000**	**12, − 104, 14**	**L. Occipital Lobe (BA19)**
5.14	0.000	6, − 56, 32	R. Precuneus
4.83	0.000	− 44, − 2, − 18	L. sup. temporal gyrus (BA22)
4.82	0.000	− 8, 60, 36	L. medial frontal gyrus (BA10)
3.89	0.000	52, − 8, − 2	R. sup. temporal gyrus (BA22)
3.09	0.01⁎	− 28, − 12, − 22	L. Hippocampus
2.37	0.01⁎	24, − 4, − 22	R. Hippocampus
Left HS	**3.33**	**0.000**	**50, − 12, 6**	**R. sup. temporal gyrus (BA22)**
3.29	0.000	− 2, 56, − 12	L. medial frontal gyrus (BA10)
3.16	0.001	22, − 12, − 24	R. Hippocampus
3.03	0.001	6, − 46, 36	R. Precuneus
Right HS	**5.63**	**0.000**	**12, − 104, 6**	**R. Occipital Lobe (BA19)**
5.18	0.000	− 4, − 64, 30	L. Precuneus
3.35	0.000	− 2, 66, 8	L. medial frontal gyrus (BA10)
3.35	0.000	− 46, − 18, − 2	L. sup. temporal gyrus (BA22)
2.84	0.002⁎	− 28, − 20, − 20	L. Hippocampus
Left HS < Controls	**3.32**	**0.029**⁎⁎	**− 8, − 12, 48**	**L. Hippocampus**
Right HS < Controls	**2.42**	**0.066**⁎⁎	**26, − 4, − 22**	**R. Amygdala**

HS = Hippocampal Sclerosis; MNI = Montreal Neurological Institute; BA = Brodmann Area; L = left; R = right.

⁎ p < 0.01 uncorrected.

⁎⁎ p < 0.05 Family-Wise Error corrected after 8 mm sphere small volume correction based on peak activation.

**Table 3:** Mean performance measures and significance values for each group in all tasks.

Performance measures	Left HS		Right HS		Controls		p. value
M	S.D	M	S.D	M	S.D
Digit span backwards	3.6	1.2	3.1	.89	4.5	.83	.001⁎
Gesture span	2.6	.65	2.7	.73	3.1	1.81	.424
Motor sequences	4.31	2	3.84	2.4	6	1.81	.131
2-back % correct	51.2	21.4	56.8	23.4	68.1	21.1	.399
PCA composite	−.256	.83	−.305	1.02	.711	083	.049⁎

M = mean; S.D = Standard Deviation; HS = Hippocampal Sclerosis. Age was included as a nuisance variable in all analyses.

⁎ Significant group difference p < 0.05 (ANOVA).

## References

[bb0005] Abrahams S., Morris R.G., Polkey C.E. (1999). Hippocampal involvement in spatial and working memory: a structural MRI analysis of patients with unilateral mesial temporal lobe sclerosis. Brain Cogn..

[bb0010] Anticevic A., Repovs G., Shulman G.L., Barch D.M. (2010). When less is more: TPJ and default network deactivation during encoding predicts working memory performance. Neuroimage.

[bb0015] Astur R.S., Constable R.T. (2004). Hippocampal dampening during a relational memory task. Behav. Neurosci..

[bb0315] Astur R.S., St Germain S.A., Baker E.K., Calhoun V., Pearlson G.D., Constable R.T. (2005). fMRI hippocampal activity during a virtual radial arm maze. Appl. Psychophysiol. Biofeedback.

[bb0025] Axmacher N., Mormann F., Fernandez G., Cohen M.X., Elger C.E., Fell J. (2007). Sustained neural activity patterns during working memory in the human medial temporal lobe. J. Neurosci..

[bb0030] Axmacher N., Schmitz D.P., Wagner T., Elger C.E., Fell J. (2008). Interactions between medial temporal lobe, prefrontal cortex, and inferior temporal regions during visual working memory: a combined intracranial EEG and functional magnetic resonance imaging study. J. Neurosci..

[bb0035] Axmacher N., Elger C.E., Fell J. (2009). Working memory-related hippocampal deactivation interferes with long-term memory formation. J. Neurosci..

[bb0040] Baddeley A. (2000). The episodic buffer: a new component of working memory?. Trends Cogn. Sci..

[bb0045] Black L.C., Schefft B.K., Howe S.R., Szaflarski J.P., Yeh H.S., Privitera M.D. (2010). The effect of seizures on working memory and executive functioning performance. Epilepsy Behav..

[bb0050] Bonelli S.B., Powell R.H., Yogarajah M. (2010). Imaging memory in temporal lobe epilepsy: predicting the effects of temporal lobe resection. Brain.

[bb0060] Callicott J.H., Mattay V.S., Bertolino A. (1999). Physiological characteristics of capacity constraints in working memory as revealed by functional MRI. Cereb. Cortex.

[bb0320] Campo P., Garrido M.I., Moran R.J., Maestu F., Garcia-Morales I., Gil-Nagel A., Del Pozo F., Dolan R., Friston K.J. (2011). Remote effects of hippocampal sclerosis on effective connectivity during working memory encoding: a case of connectional diaschisis?. Cereb. Cortex.

[bb0070] Canavan A.G., Passingham R.E., Marsden C.D., Quinn N., Wyke M., Polkey C.E. (1989). Sequence ability in parkinsonians, patients with frontal lobe lesions and patients who have undergone unilateral temporal lobectomies. Neuropsychologia.

[bb0075] Cashdollar N., Malecki U., Rugg-Gunn F.J., Duncan J.S., Lavie N., Duzel E. (2009). Hippocampus-dependent and -independent theta-networks of active maintenance. Proc. Natl. Acad. Sci. U. S. A..

[bb0080] Cashdollar N., Duncan J.S., Duzel E. (2011). Challenging the classical distinction between long-term and short-term memory: reconsidering the role of the hippocampus. Future Neurol..

[bb0085] Cave C.B., Squire L.R. (1992). Intact verbal and nonverbal short-term memory following damage to the human hippocampus. Hippocampus.

[bb0090] Corcoran R., Upton D. (1993). A role for the hippocampus in card sorting?. Cortex.

[bb0095] Cousijn H., Rijpkema M., Qin S., van Wingen G.A., Fernández G. (2012). Phasic deactivation of the medial temporal lobe enables working memory processing under stress. Neuroimage.

[bb0100] Fuentemilla L., Penny W.D., Cashdollar N., Bunzeck N., Düzel E. (2010). Theta-coupled periodic replay in working memory. Curr. Biol..

[bb0105] Gevins A., Cutillo B. (1993). Spatiotemporal dynamics of component processes in human working memory. Electroencephalogr. Clin. Neurophysiol..

[bb0110] Grippo A., Pelosi L., Mehta V., Blumhardt L.D. (1996). Working memory in temporal lobe epilepsy: an event-related potential study. Electroencephalogr. Clin. Neurophysiol..

[bb0115] Hampson M., Driesen N.R., Skudlarski P., Gore J.C., Constable R.T. (2006). Brain connectivity related to working memory performance. J. Neurosci..

[bb0120] Hermann B.P., Wyler A.R., Richey E.T. (1988). Wisconsin Card Sorting Test performance in patients with complex partial seizures of temporal-lobe origin. J. Clin. Exp. Neuropsychol..

[bb0125] Jayakar P., Bernal B., Santiago Medina L., Altman N. (2002). False lateralization of language cortex on functional MRI after a cluster of focal seizures. Neurology.

[bb0130] Jeneson A., Squire L.R. (2011). Working memory, long-term memory, and medial temporal lobe function. Learn. Mem..

[bb0135] Karlsgodt K.H., Shirinyan D., van Erp T.G., Cohen M.S., Cannon T.D. (2005). Hippocampal activations during encoding and retrieval in a verbal working memory paradigm. Neuroimage.

[bb0140] Keller S.S., Roberts N. (2008). Voxel-based morphometry of temporal lobe epilepsy: an introduction and review of the literature. Epilepsia.

[bb0325] Kim S.Y., Lee H.W., Jung D.K., Suh C.K., Park S.P. (2006). Cognitive effects of low-dose topiramate compared with oxcarbazepine in epilepsy patients. J. Clin. Neurol..

[bb0150] Koenigs M., Barbey A.K., Postle B.R., Grafman J. (2009). Superior parietal cortex is critical for the manipulation of information in working memory. J. Neurosci..

[bb0155] Krauss G.L., Summerfield M., Brandt J., Breiter S., Ruchkin D. (1997). Mesial temporal spikes interfere with working memory. Neurology.

[bb0160] Kumari V., Gray J.A., Ffytche D.H. (2003). Cognitive effects of nicotine in humans: an fMRI study. Neuroimage.

[bb0165] Labate A., Cerasa A., Aguglia U., Mumoli L., Quattrone A., Gambardella A. (2011). Neocortical thinning in “benign” mesial temporal lobe epilepsy. Epilepsia.

[bb0170] Liao W., Zhang Z., Pan Z. (2011). Default mode network abnormalities in mesial temporal lobe epilepsy: a study combining fMRI and DTI. Hum. Brain Mapp..

[bb0175] Mainy N., Kahane P., Minotti L., Hoffmann D., Bertrand O., Lachaux J.P. (2007). Neural correlates of consolidation in working memory. Hum. Brain Mapp..

[bb0180] Meyer-Lindenberg A., Poline J.B., Kohn P.D., Holt J.L., Egan M.F., Weinberger D.R., Berman K.F. (2001). Evidence for abnormal cortical functional connectivity during working memory in schizophrenia. Am. J. Psychiatry.

[bb0185] Meyer-Lindenberg A.S., Olsen R.K., Kohn P.D., Brown T., Egan M.F., Weinberger D.R., Berman K.F. (2005). Regionally specific disturbance of dorsolateral prefrontal–hippocampal functional connectivity in schizophrenia. Arch. Gen. Psychiatry.

[bb0190] Nee D.E., Jonides J. (2008). Neural correlates of access to short-term memory. Proc. Natl. Acad. Sci. U. S. A..

[bb0195] Nee D.E., Jonides J. (2011). Dissociable contributions of prefrontal cortex and the hippocampus to short-term memory: evidence for a 3-state model of memory. Neuroimage.

[bb0200] Nelson H.E., Willison J.R. (1991). The Revised National Adult Reading Test — Test Manual.

[bb0205] Oberauer K. (2002). Access to information in working memory: exploring the focus of attention. J. Exp. Psychol. Learn. Mem. Cogn..

[bb0210] Olsen R.K., Nichols E.A., Chen J., Hunt J.F., Glover G.H., Gabrieli J.D., Wagner A.D. (2009). Performance-related sustained and anticipatory activity in human medial temporal lobe during delayed match-to-sample. J. Neurosci..

[bb0220] Owen A.M. (2000). The role of the lateral frontal cortex in mnemonic processing: the contribution of functional imaging. Exp. Brain Res..

[bb0215] Owen A.M., Morris R.G., Sahakian B.J., Polkey C.E., Robbins T.W. (1996). Double dissociations of memory and executive functions in working memory tasks following frontal lobe excisions, temporal lobe excisions or amygdalo-hippocampectomy in man. Brain.

[bb0225] Owen A.M., McMillan K.M., Laird A.R., Bullmore E. (2005). N-back working memory paradigm: a meta-analysis of normative functional neuroimaging studies. Hum. Brain Mapp..

[bb0230] Poch C., Fuentemilla L., Barnes G.R., Düzel E. (2011). Hippocampal theta-phase modulation of replay correlates with configural-relational short-term memory performance. J. Neurosci..

[bb0235] Raichle M.E., MacLeod A.M., Snyder A.Z., Powers W.J., Gusnard D.A., Shulman G.L. (2001). A default mode of brain function. Proc. Natl. Acad. Sci. U. S. A..

[bb0240] Ranganath C., Blumenfeld R.S. (2005). Doubts about double dissociations between short- and long-term memory. Trends Cogn. Sci..

[bb0245] Riley J.D., Franklin D.L., Choi V. (2010). Altered white matter integrity in temporal lobe epilepsy: association with cognitive and clinical profiles. Epilepsia.

[bb0250] Schon K., Quiroz Y.T., Hasselmo M.E., Stern C.E. (2009). Greater working memory load results in greater medial temporal activity at retrieval. Cereb. Cortex.

[bb0255] Shrager Y., Levy D.A., Hopkins R.O., Squire L.R. (2008). Working memory and the organization of brain systems. J. Neurosci..

[bb0260] Smith M.E., Gevins A., McEvoy L.K., Meador K.J., Ray P.G., Gilliam F. (2006). Distinct cognitive neurophysiologic profiles for lamotrigine and topiramate. Epilepsia.

[bb0265] Squire L.R. (1992). Memory and the Hippocampus: a synthesis from findings in rats, monkeys and humans. Psychol. Rev..

[bb0270] Stretton J., Thompson P.J. (2012). Frontal lobe function in temporal lobe epilepsy. Epilepsy Res..

[bb0330] Talmi D., Grady C.L., Goshen-Gottstein Y., Moscovitch M. (2005). Neuroimaging the serial position curve. A test of single-store versus dual-store models. Psychol. Sci..

[bb0335] Tudesco Ide S., Vaz L.J., Mantoan M.A., Belzunces E., Noffs M.H., Caboclo L.O., Yacubian E.M., Sakamoto A.C., Bueno O.F. (2010). Assessment of working memory in patients with mesial temporal lobe epilepsy associated with unilateral hippocampal sclerosis. Epilepsy Behav..

[bb0285] Vlooswijk M.C., Jansen J.F., Jeukens C.R., Majoie M., Hofman P.A., De Krom M.C., Aldenkamp A.P., Backes W.H. (2011). Memory processes and prefrontal network dysfunction in cryptogenic epilepsy. Epilepsia.

[bb0290] Vollmar C., O'Muicheartaigh J., Barker G.J., Symms M.R., Thompson P., Kumari V., Duncan J.S., Janz D., Richardson M.P., Koepp M.J. (2011). Motor system hyperconnectivity in juvenile myoclonic epilepsy: a cognitive functional magnetic resonance imaging study. Brain.

[bb0295] Wagner D.D., Sziklas V., Garver K.E., Jones-Gotman M. (2009). Material-specific lateralization of working memory in the medial temporal lobe. Neuropsychologia.

[bb0300] Wechsler D. (1997). Manual for the Wechsler Adult Intelligence Scale-III.

[bb0305] Woermann F.G., Barker G.J., Birnie K.D., Meencke H.J., Duncan J.S. (1998). Regional changes in hippocampal T2 relaxation and volume: a quantitative magnetic resonance imaging study of hippocampal sclerosis. J. Neurol. Neurosurg. Psychiatry.

[bb0310] Zarahn E., Rakitin B., Abela D., Flynn J., Stern Y. (2005). Positive evidence against human hippocampal involvement in working memory maintenance of familiar stimuli. Cereb. Cortex.

